# Cisplatin and Pemetrexed Activate AXL and AXL Inhibitor BGB324 Enhances Mesothelioma Cell Death from Chemotherapy

**DOI:** 10.3389/fphar.2017.00970

**Published:** 2018-01-11

**Authors:** Derek B. Oien, Tamás Garay, Sarah Eckstein, Jeremy Chien

**Affiliations:** ^1^Division of Molecular Medicine, Department of Internal Medicine, UNMHSC School of Medicine, University of New Mexico Comprehensive Cancer Center, Albuquerque, NM, United States; ^2^Second Department of Pathology, Semmelweis University, Budapest, Hungary; ^3^Department of Cancer Biology, University of Kansas Medical Center, Kansas City, KS, United States

**Keywords:** AXL, reactive oxygen species, cisplatin, pemetrexed, mesothelioma, BGB324, chemotherapy resistance

## Abstract

Reactive oxygen species (ROS) can promote or inhibit tumorigenesis. In mesothelioma, asbestos exposure to serous membranes induces ROS through iron content and chronic inflammation, and ROS promote cell survival signaling in mesothelioma. Moreover, a current chemotherapy regimen for mesothelioma consisting of a platinum and antifolate agent combination also induce ROS. Mesothelioma is notoriously chemotherapy-resistant, and we propose that ROS induced by cisplatin and pemetrexed may promote cell survival signaling pathways, which ultimately may contribute to chemotherapy resistance. In The Cancer Genome Atlas datasets, we found AXL kinase expression is relatively high in mesothelioma compared to other cancer samples. We showed that ROS induce the phosphorylation of AXL, which was blocked by the selective inhibitor BGB324 in VMC40 and P31 mesothelioma cells. We also showed that cisplatin and pemetrexed induce the phosphorylation of AXL and Akt, which was also blocked by BGB324 as well as by N-acetylcysteine antioxidant. AXL knockdown in these cells enhances sensitivity to cisplatin and pemetrexed. Similarly, AXL inhibitor BGB324 also enhances sensitivity to cisplatin and pemetrexed. Finally, higher synergy was observed when cells were pretreated with BGB324 before adding chemotherapy. These results demonstrate cisplatin and pemetrexed induce ROS that activate AXL, and blocking AXL activation enhances the efficacy of cisplatin and pemetrexed. These results suggest AXL inhibition combined with the current chemotherapy regimen may represent an effective strategy to enhance the efficacy of chemotherapy in mesothelioma. This is the first study, to our knowledge, on chemotherapy-induced activation of AXL and cell survival pathways associated with ROS signaling.

## Introduction

Cellular oxidative stress is characterized by elevated reactive oxygen species (ROS), and is a frequent phenotype of cancer cells (Szatrowski and Nathan, 1991; Luo et al., 2009; Oien et al., 2016). High levels of ROS can disprupt critical functions of the cell by oxidizing proteins, nucleic acids, and lipids (Oien and Moskovitz, 2008; Oien et al., 2009, 2010). These alterations are associated with both cancer initiation and tumor progression (Storz, 2005). However, elevated ROS also promote apoptosis (Watson, 2013). Several chemotherapy agents generate ROS, and increased endogenous antioxidants often correlate with a reduction in sensitivity to chemotherapy (Godwin et al., 1992; Ramanathan et al., 2005; Berndtsson et al., 2007; Hwang et al., 2015). Since cells have multiple antioxidant systems to counteract any perpetual increases in ROS, we conceptualized blocking ROS-induced cell survival signals as a method for enhancing the efficacy of current chemotherapeutic agents.

Elevated ROS levels are associated with the phases of mesothelioma progression, as well as non-surgical treatments (Benedetti et al., 2015). Malignant pleural mesothelioma (MPM) is a cancer in the serous membrane surrounding the lungs, and has a very poor survival rate (Benedetti et al., 2015). The rapid relapse of MPM tumors results in a progression-free survival median of <6 months and overall survival median of about 1 year (Bonelli et al., 2016). MPM is primarily caused by asbestos exposure (Bonelli et al., 2016). The exposure of asbestos to mesothelial cells generates ROS through iron content and chronic inflammation (Benedetti et al., 2015; Pietrofesa et al., 2016). This ROS may promote cell transformation, such as through DNA damage, and also may promote cell survival signaling (Clerkin et al., 2008; Benedetti et al., 2015). Furthermore, standard chemotherapy agents used in MPM treatment also generate ROS in addition to targeting DNA mechanisms (Berndtsson et al., 2007; Hwang et al., 2015).

The low MPM survival rate is attributed to late-stage diagnosis and resistance to chemotherapy (Benedetti et al., 2015). A platinum and antifolate drug combination are regularly used for MPM chemotherapy treatment. Cisplatin is a platinum-based drug that is activated upon entering cells, and promiscuously binds nucleic acids and proteins (Akaboshi et al., 1992; Berndtsson et al., 2007). DNA adducts caused by cisplatin correlate with cell death, but at higher concentrations cisplatin can generate ROS and cause acute apoptosis (Berndtsson et al., 2007). Pemetrexed is an antifolate drug that interferes with nucleic acid synthesis, generates ROS, and causes apoptotic cell death (Hwang et al., 2015).

MPM cells are an ideal system to investigate the paradoxical effect of ROS because ROS are implicated in the disease promotion as well as in the response to chemotherapy. ROS promote activation of several receptor tyrosine kinases (RTKs) and inhibition of associated phosphatases (Rhee et al., 2003). Several studies indicate RTKs are associated with MPM tumor progression, including epidermal growth factor receptor (EGFR) (Pache et al., 1998), vascular endothelial growth factor receptor (VEGFR) (Ohta et al., 1999; Strizzi et al., 2001), insulin growth factor 1 receptor (IGF1R) (Lee et al., 1993; Pass et al., 2004), and AXL (Ou et al., 2011). We used the data from The Cancer Genome Atlas (TCGA) to identify RTKs with a relatively high expression in MPM, and chose to further evaluate AXL. AXL promotes cell survival and proliferation (Ou et al., 2011; Wu et al., 2014). Moreover, AXL activation enhances chemotherapy resistance in lung cancer (Zhang et al., 2012). AXL pathway activates anti-apoptotic mechanisms, for example after vascular injury (Melaragno et al., 1998). AXL is activated via phosphorylation (pAXL) by ROS and growth arrest specific-6 (GAS6) (Konishi et al., 2004), and this phosphorylation can induce the PI3K/Akt pathway (Huang et al., 2013). AXL activation can be attenuated by inhibitors, and BGB324 is a first-in-class small molecule inhibitor with selectivity to AXL (Holland et al., 2010; Myers et al., 2016).

We hypothesize ROS, generated by cisplatin and pemetrexed, induce the activation of AXL and cell survival pathway, which can be blocked by AXL inhibitors to enhance the efficacy of current MPM chemotherapy drugs. We demonstrate here that cisplatin and pemetrexed generate ROS in MPM cells and induce AXL and Akt phosphorylation. Blocking AXL activation with the BGB324 inhibitor enhances sensitivity to cisplatin and pemetrexed in MPM cell lines. Finally, we show that higher sensitivity is achieved when cells were pre-treated with BGB324 4 h prior to chemotherapy.

## Materials and methods

### Reagents stock solutions

Cisplatin USP (Teva) was supplied as a 1 g/L solution in saline, and pemetrexed (Sigma) was dissolved in water immediately prior to use. BGB324 (Selleck Chemicals) was dissolved in DMSO, and diluted in water immediately prior to use. Hydrogen peroxide was diluted in water immediately prior to use from a 30 wt. % stock solution (Sigma). Solubility of all reagents were visually verified in experimental systems prior to use.

### RNA expression in TCGA samples

Data generated by the TCGA Research Network was used to compare RNA expression and ranking among datasets. Values and graphs for comparisons were obtained from cBioPortal (Cerami et al., 2012; Gao et al., 2013).

### Reverse phase protein array and cell lines

Protein lysates from MPM cells lines were subjected to a reverse phase protein array. This array was performed at the MD Anderson Functional Proteomics RPPA Core Facility. MPM cells lines used were SPC111, VMC14, VMC31, M38K, VMC40, Meso53, SPC212, and P31 (Marklund et al., 1982; Pelin-Enlund et al., 1990; Schmitter et al., 1992; Janson et al., 2008; Garay et al., 2013; Schelch et al., 2014; Hoda et al., 2016). All cell lines were maintained in Dulbecco Modified Eagle Medium (GenClone) with 10% fetal bovine serum (Sigma). Lysates were added to nitrocellulose-coated slides in five serial dilutions, and subjected to 301 antibodies. Samples probed with antibodies were analyzed by tyramide-based signal amplification approach, and relative protein levels were determined after protein loading normalization. These levels were fitted and analyzed by the SuperCurve GUI method (Hu et al., 2007). The heat map was generated in Cluster 3.0. Nonmalignant mesothelium Met-5a cells (ATCC) were used for comparing basal AXL expression to VMC40 and P31 cells, and in later cell death experiments.

### Immunoblots and antibodies

In six-well plates, 3.5 × 10^5^ cells/well incubated at 37°C for overnight attachment prior to any treatment. Proteins were extracted from cells in 2x Laemmli buffer (Bio-Rad) containing protease and phosphatase inhibitors. Except as noted, equal sample volumes were applied to SDS-PAGE gels and transferred to PVDF membranes. Membranes were incubated in Odyssey Blocking Buffer (Li-Cor) overnight at 4°C prior to probing with primary antibodies overnight at 4°C. IRDye rabbit, mouse, and goat secondary antibodies (Li-Cor) were applied to probed membranes for 45 m at room temperature, and visualized using a Li-Cor Odyssey scanning system. Primary antibodies used for standard immunoblot analysis included AXL (R&D Systems), pAktS473 and pan-Akt (Cell Signaling Technology), and beta-actin (Sigma). Based on minimal detection by standard methods, pAXLY702 (Cell Signaling Technology), GAS6 (GeneTex), and beta-actin (as system control) antibodies were used to detect corresponding protein levels by Wes capillary electrophoresis (ProteinSimple) based on the manufacturer's protocol. Chemiluminescent signal is depicted as traditional immunoblot protein bands, which are generated by Compass software (ProteinSimple), as done previously for RTKs (Furugaki et al., 2016).

### ROS detection

In six-well plates, 3.5 × 10^5^ cells/well incubated at 37°C for overnight attachment. Medium was removed from each well, and 5 μM chloromethyl-dihydrogen-2′,7′-dichlorofluorescein diacetate (CM-H_2_DCFDA) was added to each well for 20 m. After incubation, cells were lifted with trypsin (Sigma) and transferred to centrifuge tubes. Tubes were centrifuged, supernatant aspirated, and fresh medium premixed with indicated reagent(s) was added. Cells were incubated for 30 m at 37°C with gentle shaking. Prior to analysis, propidium iodide was added at a final concentration of 1.3 μg/mL to reduce dead cells during analysis through gating. Fluorescence in cells was detected with a LSR II flow cytometer (BD Biosciences) using FITC filters.

### Caspase 3 activity assay

Caspase 3 activity was measured using the DEVD-AFC (Asp-Glu-Val-Asp, 7-amino-4-trifluoromethylcoumarin) fluorescent assay as we have previously described (Bastola et al., 2016). Briefly, cells were prepared as done for immunoblots and lysed using CHAPS lysis buffer (20 mM PIPES, 100 mM NaCl, 1 mM EDTA, 0.1% (w/v) CHAPS, 10% sucrose, 10 mM DTT pH 7.2). Protein concentration was determined using the Pierce BCA Assay (Thermo Fisher). Twenty milligrams of protein was combined with 2 μL of 2 mM DEVD-AFC (Millipore) in 96-well flat-bottom plates. Two hundred microliters/well lysis buffer was added. The plate was covered and incubated at 37°C. After 2 h, fluorescence measurements were taken at Ex 400 nm and Em 510 nm using a Synergy4 plate reader (BioTek).

### AXL knockdown

VMC40 and P31 cells with AXL shRNA were generated using human GIPZ lentiviral shRNAmir individual clones (Dharmacon) according to the manufacturer's protocol. Transfected cells were selected in puromycin for 2 weeks prior to use. Knockdown was determined by immunoblots using methods described above using three different gel loading volumes for estimation of shRNA efficacy compared to nontargeting control (NTC). Cell proliferation was determined by the sulforhodamine B (SRB) assay by plating equal cells, 5,000 cells/well in 96-well plates, and incubating for 4 days. Quantification with SRB stain was done as described below, based on our previous methods (Bastola et al., 2016).

### SRB survival assays and drug synergy calculations

Short-term SRB survival assays and synergy calculations were performed as previously described (Bastola et al., 2016). Briefly, 5,000 cells were plated in 96-well plates and attached by overnight incubation. The following day, cells were treated with different concentrations of BGB324 and cisplatin or pemetrexed, and in combinations as indicated. After 72 h, cells were fixed with 10% trichloroacetic acid (Sigma). Fixed cells were washed with tap water and stained using SRB (Sigma) at room temperature for 45 m. Cells were washed with 1% acetic acid solution, and then were dissolved in 10 mM Tris, pH 10, and fluorescence measurements were taken at Ex 488 nm and Em 585 nm using a Synergy4 plate reader. Where specified, the concentration that causes 50% growth inhibition (GI_50_) was estimated using Prism software (GraphPad). Drug synergy was determined by calculating the combination indices (CIs) obtained from the fluorescence measurements. The CI values were calculated based on dividing the combination expected effect by the observed effect: CI = (D_1_ +D_2_^*^(1-D_1_))/D_observed_, where D represents the cell death from drug 1, drug 2, and the combination (observed) (Bastola et al., 2016). The mean CI values were determined from multiple drug combinations in duplicates that produced 20–80% effect from independent experiments.

### Clonogenic cell death assays

Long-term survival assays were performed as previously described (Bastola et al., 2016). Briefly, 500 cells/well were plated in 6-well plates and cells were attached by overnight incubation. Regents were added to cells and incubated for 72 h. Following treatment, medium was gently aspirated and replaced with regular growth medium. Cells were then incubated for 4–7 additional days. Once the colonies were optimal for visualization, cells were fixed with 10% trichloroacetic acid. Fixed cells were washed with tap water and stained using SRB at room temperature for 45 m. Cells were then washed with 1% acetic acid solution. The plates were air-dried, and pictures were taken using the Bio-Rad Imager System. For quantification, stained cells were dissolved in 10 mM Tris, pH 10 and the solution was transferred to 96-well plates for fluorescence measurement.

### Analyses and statistics

Densitometry values from immunoblots were determined by Image Studio software (Li-Cor). Dose-response data and non-linear regression curves were generated using Prism software. Bar charts were generated via Excel (Microsoft), and *t-test* statistical analyses used with a significance threshold of *p* < 0.05.

## Results

### AXL expression is elevated in MPM

**Figure 1:**
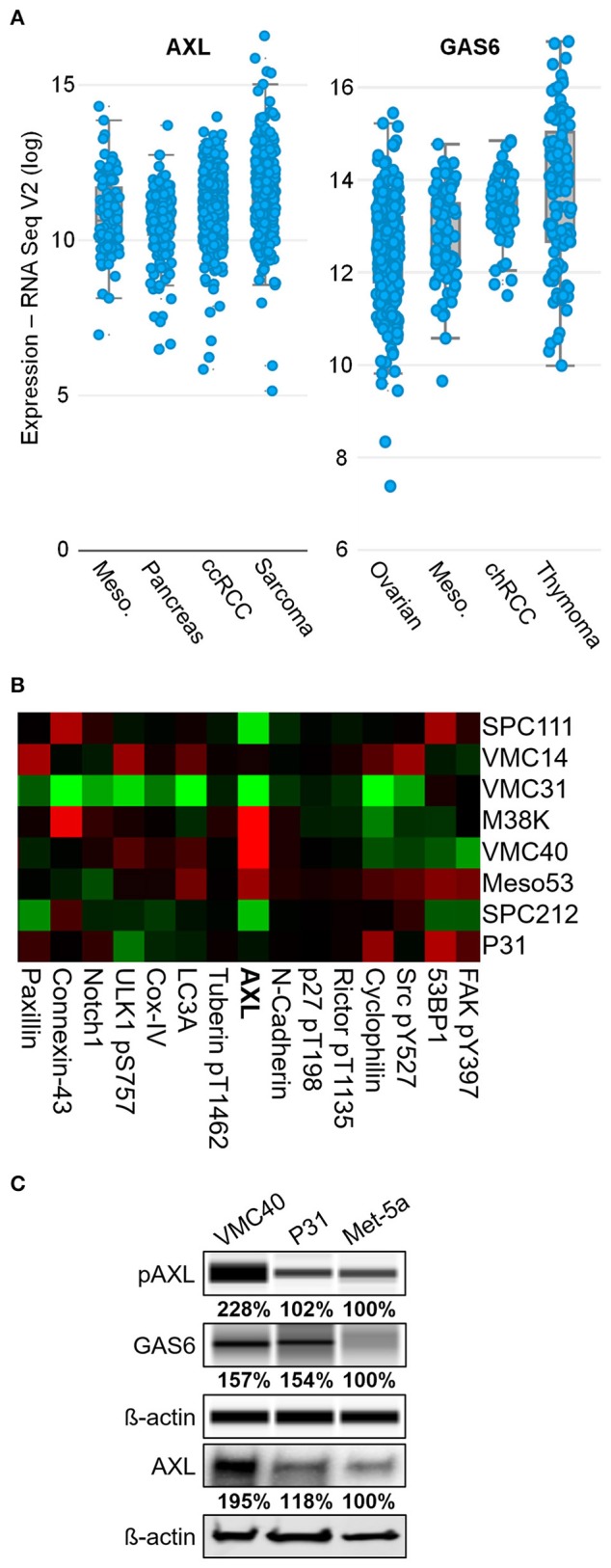
AXL is abnormally expressed in MPM. **(A)** AXL and GAS6 expression are elevated in clinical MPM samples. Of 30 TCGA cancer datasets, MPM (87 samples, *blue dots*) ranked fourth highest for AXL and third highest for GAS6 expression, and mean log_2_ values were 11.40 and 13.06, respectively. Data are RNA Seq V2 values from TCGA Research Network. **(B)** Reverse phase protein heat map subset with AXL. MPM cell extracts applied to coated slides and probed with 301 antibodies, normalized to loading. Color brightness indicates protein detection relative to median, *red* for increased and *green* for decreased detection levels. Normalized log_2_ median-centered value of AXL was 1.35 for VMC40. **(C)** Lysates analyzed for pAXL and GAS6 ligand using capillary electrophoresis, and other proteins were detected by standard immunoblot methods. Met-5a as a nonmalignant mesothelial control cell line for comparison. Densitometry values are relative to corresponding protein of Met-5a, normalized to respective ß-actin loading control.

To uncover RTKs that may be promoting MPM cell survival, the expression levels of common RTKs were ranked based on the expression among the 30 TCGA datasets accessed through the cBioPortal bioinformatics web tool (Cerami et al., 2012; Gao et al., 2013). AXL expression was the fourth highest in MPM samples among other cancer types, with a mean log_2_ value of 11.40 (Figure 1A and Supplementary Figure 1). This expression value for AXL was higher than IGF1R (11.04), EGFR (10.14), and the other “TAM” kinases Tyro3 and Mer. The VEGFR1 kinase expression was higher than AXL in MPM, however, the VEGF ligand (*VEGFA* gene) was not expressed in MPM as high as most other cancers in the dataset (ranking #20 of 30). The AXL ligand, GAS6, had the third highest expression in MPM, versus the other cancer datasets, with a mean log_2_ value of 13.06 (Figure 1A and Supplementary Figure 2). Moreover, AXL was demonstrated in prior studies to regulate MPM proliferation (Ou et al., 2011), and ROS was shown to activate AXL and increase GAS6 binding (Konishi et al., 2004). Based on literature, and AXL and GAS6 expression in MPM, the AXL kinase was selected for further study.

Eight MPM cell lines were used in a reverse phase protein array to detect differences in protein expression including AXL (Figure 1B and Supplementary Figure 3). Of these cell lines, AXL expression was highest in VMC40 cells (normalized log_2_ median-centered value of 1.35), and P31 cells had AXL expression near the median (−0.07). The immunoblot analysis confirmed that AXL expression and phosphorylation were higher in VMC40 than the nonmalignant mesothelial cell line Met-5a (Figure 1C). In addition, GAS6 expression was also higher in VMC40 than in Met-5a cells. These results suggest autocrine AXL pathway is active in MPM cells.

### Cisplatin and pemetrexed generate ROS

**Figure 2:**
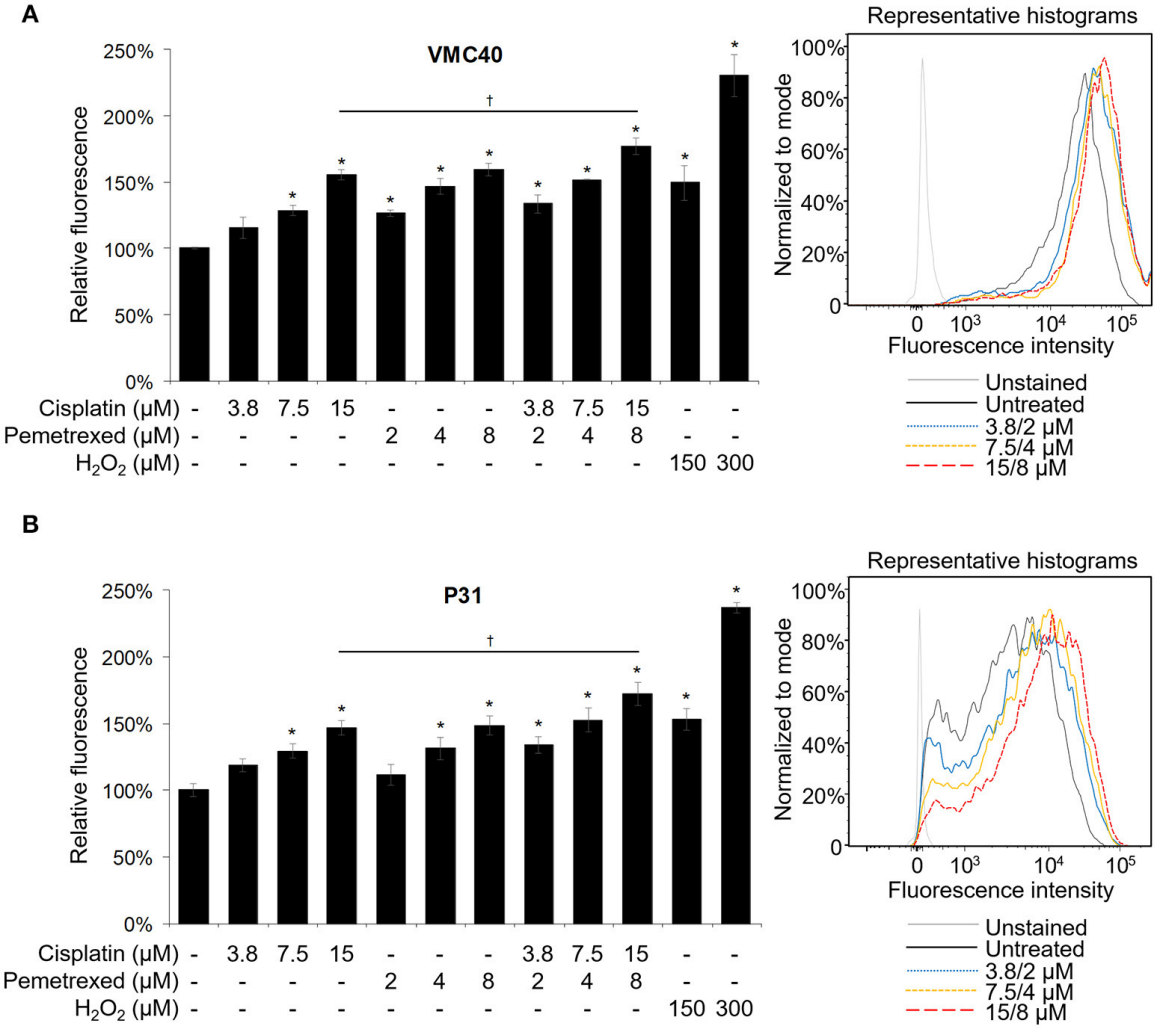
Cisplatin and pemetrexed induce ROS generation. The CM-H_2_DCFDA ROS indicator was preloaded into **(A)** VMC40 and **(B)** P31 cell lines, followed by 30 m of cisplatin and pemetrexed treatment. Fluorescent signal was detected by flow cytometry, and area-under-curve values were compared to untreated samples as percentages. Representative histograms of combined cisplatin and pemetrexed concentrations on *right*. Error bars represent ±SEM of three experiments. ^*^ and † denote *p* < 0.05 via *t*-test for value compared to untreated control and to drug combination, respectively.

Cisplatin and pemetrexed each have been shown previously to induce elevated ROS in cells (Berndtsson et al., 2007; Hwang et al., 2015), but have not been evaluated for cellular ROS increases as a combination. Cisplatin can induce detectable ROS increases in melanoma cells at concentrations of 10 and 20 μM, measured at 3 h (Berndtsson et al., 2007). However, at MPM clinical doses (e.g., 75 mg/m^2^) plasma concentrations of cisplatin is below 20 μM (Andersson et al., 1996). In contrast, MPM pemetrexed doses of 500 mg/m^2^ can achieve C_max_ values near 250 μM (Sweeney et al., 2006). Moreover, pemetrexed could induce ROS at a concentration of 2 μM in MPM cells, measured at 24 h (Hwang et al., 2015). In both VMC40 and P31 cells, higher concentrations of cisplatin (7.5 and 15 μM), multiple concentrations of pemetrexed (2, 4, and 8 μM), and multiple concentrations of combined cisplatin and pemetrexed (3.8/2, 7.5/4, and 15/8 μM, respectively) induced significant increases in ROS after 30 m versus control cells when using the CM-H_2_DCFDA indicator (Figure 2). The combination of 15 μM cisplatin and 8 μM pemetrexed produces significantly higher ROS than cisplatin alone (*p* = 0.049 and 0.047 for VMC40 and P31, respectively), but not significantly different from pemetrexed alone.

### Cisplatin and pemetrexed can activate AXL

**Figure 3:**
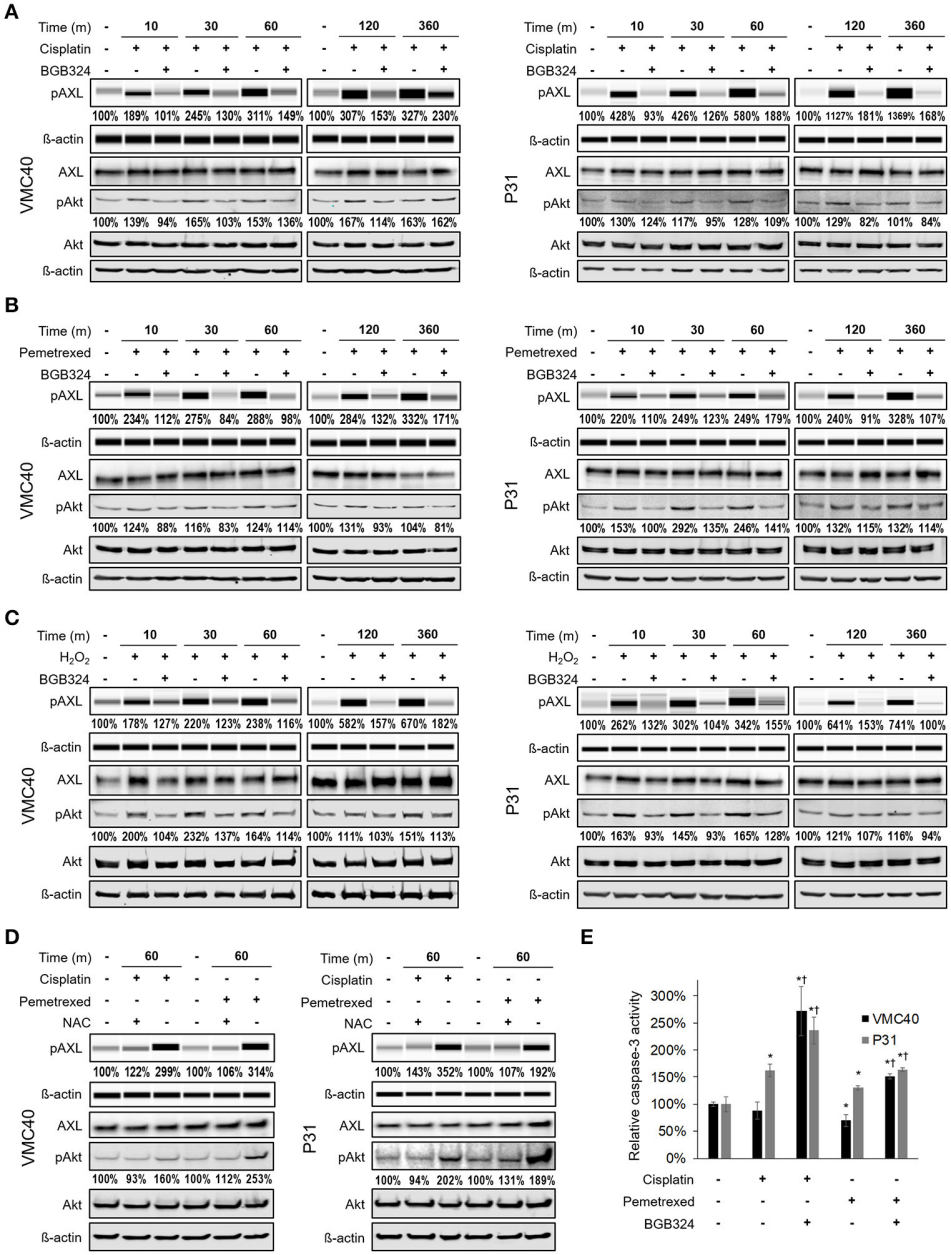
Cisplatin and pemetrexed promote phosphorylation of AXL and downstream Akt. **(A–C)** Time course of pAXL and downstream pAkt detection in the presence of **(A)** 15 μM cisplatin, **(B)** 8 μM pemetrexed, and **(C)** 150 μM H_2_O_2_. Phosphorylation of AXL was inhibited by adding 2 μM BGB324. Densitometry values as percentage of untreated control are shown for pAXL and pAkt, normalized to respective beta-actin. **(D)** pAXL detection with the addition of 4 mM N-acetylcysteine (NAC) antioxidant at 60 m. Lysates **(A–D)** were analyzed for pAXL using capillary electrophoresis, and other proteins were detected by standard immunoblot methods. Densitometry values are relative to corresponding protein of untreated control, normalized to respective ß-actin loading control. **(E)** Caspase 3 activity after 24 h of indicated drug treatment. Error bars represent ±SEM of three experiments. ^*^ and † denote *p* < 0.05 via *t*-test for value compared to untreated control and to drug combination, respectively.

Prior studies have shown AXL can be phosphorylated by hydrogen peroxide (Konishi et al., 2004), but it has not been evaluated if chemotherapy agents can activate AXL. Based on our observation that chemotherapy agents increase ROS levels (Figure 2), VMC40 and P31 MPM cells were treated with 15 μM cisplatin and 8 μM pemetrexed for short (minutes) and relatively long (6 h) time points. Increases in AXL phosphorylation were detected after 10 m of exposure to cisplatin and pemetrexed (Figures 3A,B, Supplementary Figure 4A). Consistent with prior studies indicating that AXL inhibitor BGB324 attenuates ROS-induced phosphorylation of AXL (Smolock and Korshunov, 2010), AXL phosphorylation was blocked when MPM cells were co-treated with BGB324. Again, consistent with prior studies indicating that pro-survival kinase Akt1 was activated downstream of AXL (Huang et al., 2013), both cisplatin and pemetrexed induce Akt phosphorylation of S473 at variable time points. Akt phosphorylation was blocked by BGB324. The pAkt levels did not change from adding BGB324 inhibitor alone compared to no treatment (Supplementary Figure 4B), suggesting that BGB324 does not inhibit Akt directly. In parallel, cells treated with 150 μM hydrogen peroxide show AXL and Akt phosphorylation, and this phosphorylation was blocked by BGB324 (Figure 3C). Cells co-treated with 4 mM N-acetylcysteine antioxidant did not show an increase in AXL and Akt phosphorylation (Figure 3D). Collectively, these results suggest cisplatin and pemetrexed induce ROS which then activate AXL and downstream Akt. Finally, this signaling cascade can be blocked by BGB324 or N-acetylcysteine.

Based on the phosphorylation of Akt, we next determined changes in downstream caspase-3 activity with and without BGB324 after 24 h of chemotherapy exposure (Figure 3E). The presence of BGB324 significantly increased the caspase-3 activity in cells treated with cisplatin and pemetrexed. Overall, these results indicate the possibility that BGB324 could enhance the cytotoxicity of these chemotherapy agents in VMC40 and P31.

### AXL knockdown enhances cisplatin and pemetrexed sensitivity

**Figure 4:**
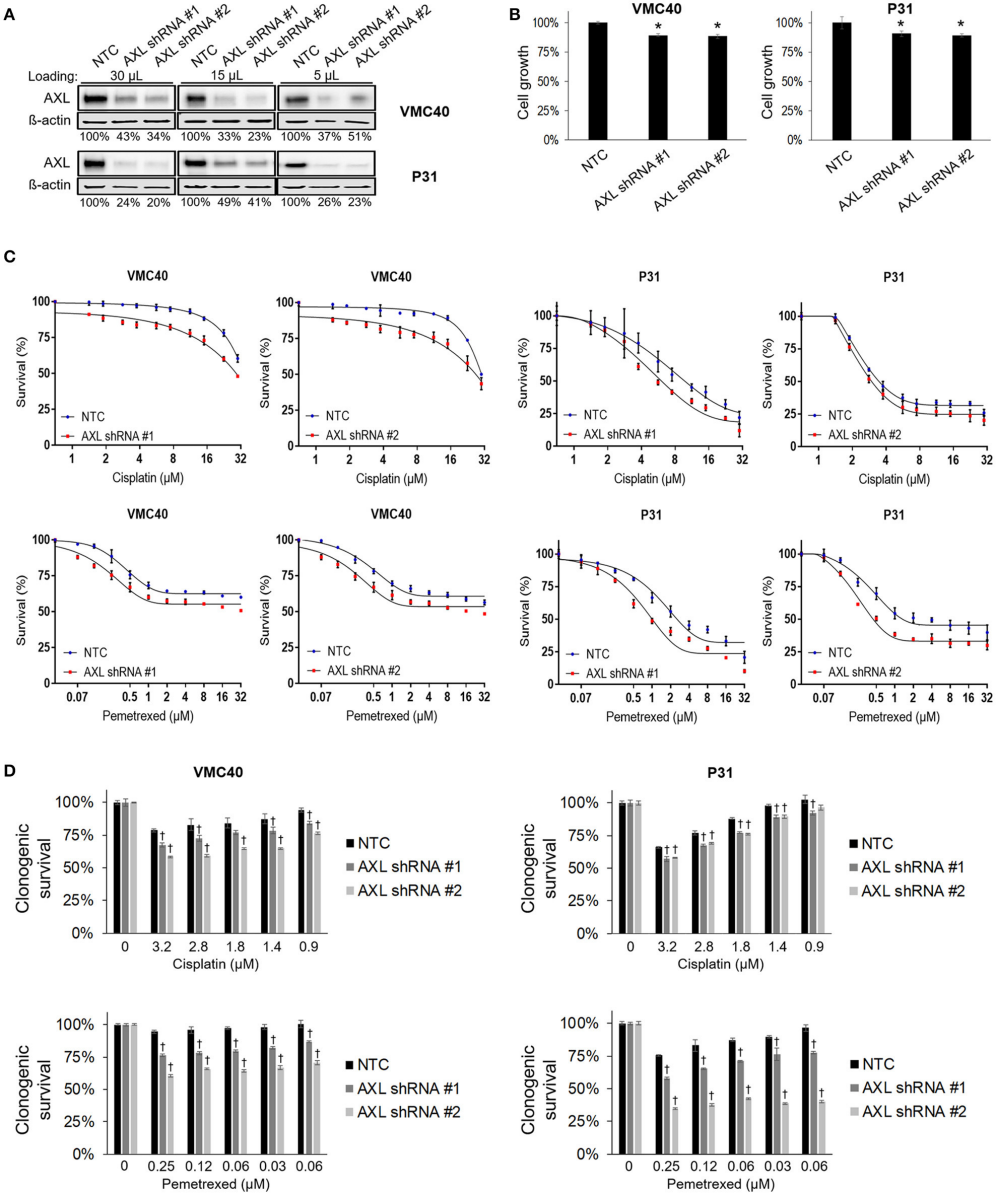
AXL knockdown enhances cisplatin and pemetrexed efficacy. **(A)** Relative quantification of AXL after shRNA AXL knockdown using three gel loading volumes of 5, 15, and 30 μL. Values are percentage of NTC. **(B)** Cell proliferation via SRB assay, values compared as percentage to NTC after 4 days of plating equal cell numbers. ^*^Denotes *p* < 0.05 via *t*-test for value compared to NTC value. **(C)** Cell survival percentages by SRB assay after 72 h treatment with cisplatin (top) and pemetrexed (bottom) at incrementing concentrations. **(D)** Cell survival percentages by clonogenic assay after 72 h treatment with cisplatin (top) and pemetrexed (bottom) at incrementing concentrations. Values are relative to no drug treatment (100% for both NTC and AXL shRNA). †Denotes *p* < 0.05 via *t*-test for value compared to NTC value at the same concentration. Error bars represent ±SEM of three experiments (B-D).

Based on the results that cisplatin and pemetrexed enhance pAXL and pAkt (Figure 3), we hypothesized AXL inhibition may enhance sensitivity to these chemotherapy agents. AXL was knocked down via AXL shRNA in both VMC40 and P31 cells (Figure 4A). AXL knockdown efficiency was estimated by immunoblots using loading volumes of 5, 15, and 30 μL. Detected AXL of VMC40 cells averaged 37.7% for shRNA#1 and 36.1% for shRNA#2 compared to NTC controls, and of P31 cells averaged 33.0% for shRNA#1 and 28.4% for shRNA#2. The AXL shRNA decreased cell proliferation (Figure 4B). Using the SRB assay and comparing to NTC, cells with AXL shRNA were more sensitive to cell death at increasing concentrations of cisplatin and pemetrexed (Figure 4C, Supplementary Figure 5). To analyze the drug response following AXL knockdown, drug response was compared at each concentration respective to the absence of drug. A significant decrease in cell survival was observed in AXL knockdown cells compared to NTC at 1.88 and 15 μM for cisplatin in VMC40 cells, and at 1 and 8 μM for pemetrexed in both VMC40 and P31 cells (Supplementary Figure 5). Consequently, we verified these results using secondary long-term clonogenic assays where the drug is washed out after 72 h (Figure 4D and Supplementary Figure 6). These data suggest AXL may be targeted to enhance sensitivity to cisplatin and pemetrexed.

### Blocking AXL activation enhances cisplatin and pemetrexed efficacy

**Figure 5:**
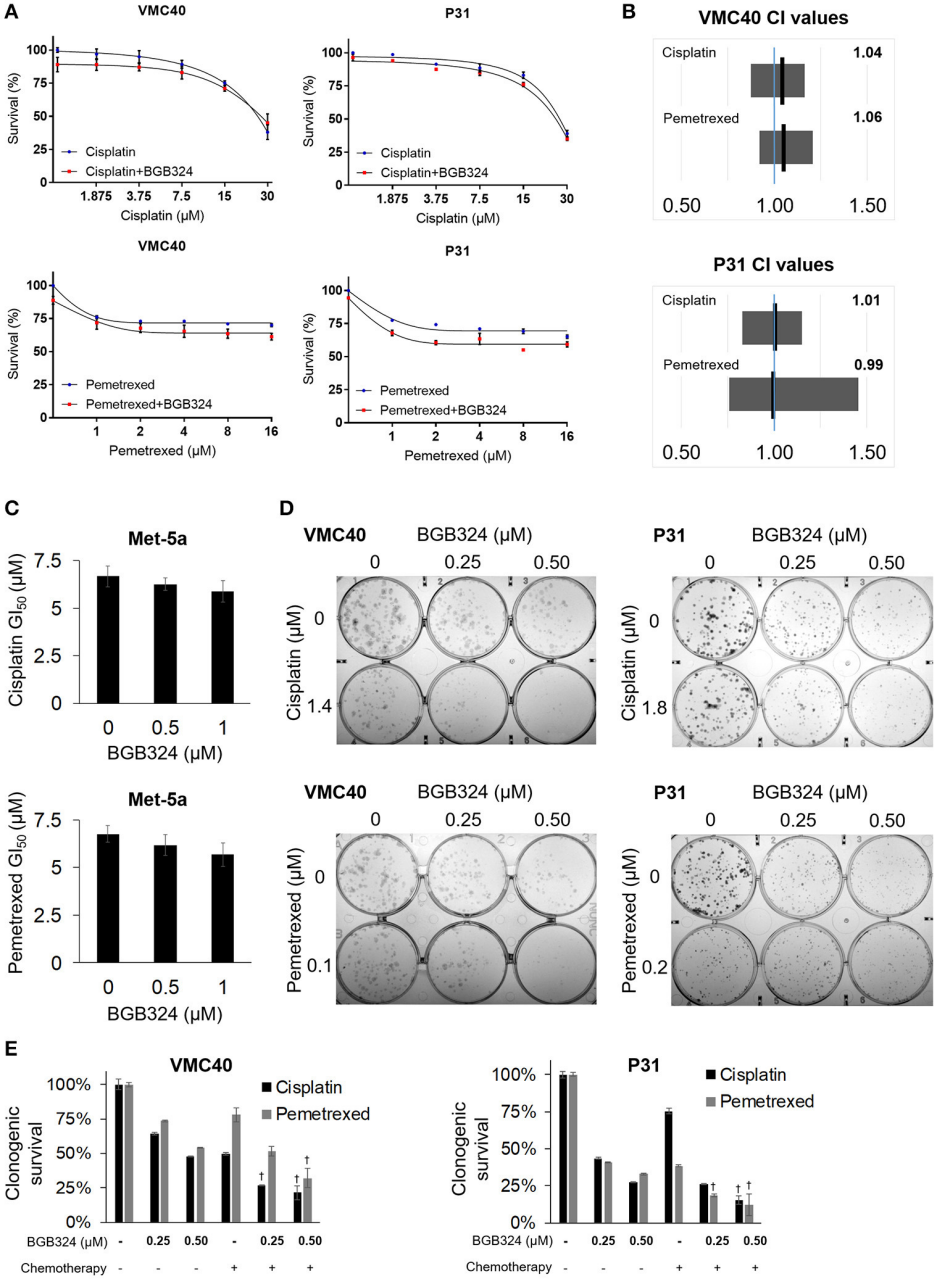
Combination of BGB324 with cisplatin and pemetrexed enhances MPM cell death. **(A)** Cell survival for incrementing concentrations of cisplatin and pemetrexed with and without 0.5 μM BGB324. Values determined by SRB assay at 72 h. Actual values, dose-response curve of BGB324, and statistical analysis at specified concentrations are shown in Supplementary Figure 7. **(B)** CI range (gray box) and mean (black line) from full data shown in Supplementary Figure 7A. The reference value, 1.00, is indicated by a blue line. CI < 1.00 is considered synergistic. CI values calculated as described in section Materials and Methods. **(C)** GI_50_ values for cisplatin and pemetrexed in nonmalignant Met-5a cells. **(D)** Representative images of clonogenic assays after 72 h treatment and then allowing cells to form colonies in absence of drugs. **(E)** Quantification of clonogenic assays. †Denotes *p* < 0.05 via *t*-test for value compared to values of single agents. Error bars represent ±SEM of three experiments **(A,C,E)**.

Building upon the observation that AXL knockdown enhances sensitivity to cisplatin and pemetrexed, we next evaluated if inhibiting AXL phosphorylation would result in enhanced sensitivity to cisplatin and pemetrexed. The selective AXL inhibitor, BGB324, has been studied as a monotherapy and with other chemotherapeutic agents (Holland et al., 2010; Wnuk-Lipinska et al., 2014; Wang et al., 2016). We used the SRB assay to determine the extent of cytotoxicity induced by BGB324 in the presence of incrementing concentrations of cisplatin and pemetrexed (Figure 5A and Supplementary Figure 7). In these SRB assays, combined effects of BGB324 with cisplatin and pemetrexed were mostly additive with CI values of approximately 1 (Figure 5B). CI values <1 indicate synergistic effects, while values >1 indicate antagonistic effects. In contrast, the addition of BGB324 did not have a significant effect on GI_50_ values with incrementing concentrations of cisplatin and pemetrexed in nonmalignant Met-5a cells (Figure 5C). To further validate results from VMC40 and P31 SRB assays, we performed clonogenic assays in these cell lines (Figures 5D,E). Both cell lines showed increased cell death at relatively low concentrations compared to SRB assays, suggesting a fraction of cells surviving in SRB assays could be in senescence. Moreover, mean CIs from the clonogenic assay were 0.95 for 1.4 μM cisplatin with BGB324 and 0.91 for 0.1 μM pemetrexed with BGB324 in VMC40 cells, which suggests marginal synergistic cytotoxicity. For P31 cells, the mean CIs for concentrations shown were 0.93 for cisplatin with BGB324 and 1.02 for pemetrexed with BGB324.

**Figure 6:**
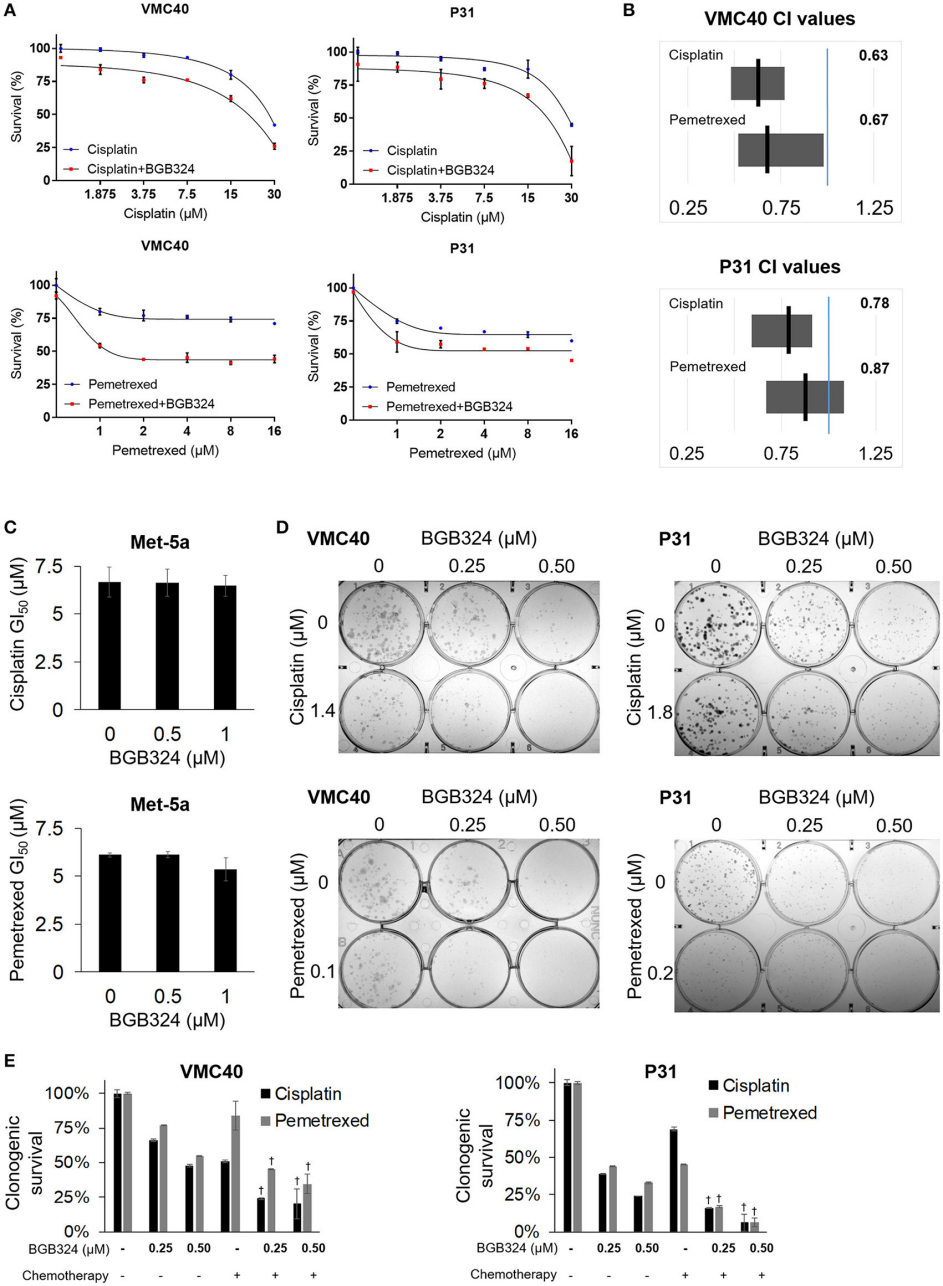
Time-sequenced BGB324 combination with cisplatin and pemetrexed enhances MPM cell death. BGB324 was added 4 h before other drugs. **(A)** Cell survival for incrementing concentrations of cisplatin and pemetrexed with and without 0.5 μM BGB324. Values determined by SRB assay at 72 h. Actual values, incrementing concentrations of BGB324, and statistical analysis at specified concentrations are shown in Supplementary Figure 8. **(B)** CI range (gray box) and mean (black line) from full data shown in Supplementary Figure 8A. The reference value, 1.00, is indicated by a blue line. CI < 1.00 is considered synergistic. CI values calculated as described in section Materials and Methods. **(C)** GI_50_ values for cisplatin and pemetrexed in nonmalignant Met-5a cells. **(D)** Representative images of clonogenic assays after 72 h treatment and then allowing cells to form colonies in absence of drugs. **(E)** Quantification of clonogenic assays. †Denotes *p* < 0.05 via *t*-test for value compared to values of single agents. Error bars represent ±SEM of three experiments **(A,C,E)**.

The mainly additive drug-drug interaction between BGB324 and cisplatin or pemetrexed raises the question if the sequence of drug exposure must be optimized. We performed essentially the same assays in Figure 5, except the BGB324 inhibitor was added 4 h prior to any addition of cisplatin and pemetrexed (Figure 6). In SRB assays, cell death was enhanced when pre-treatment of BGB324 was added to incrementing concentrations of cisplatin and pemetrexed (Figure 6A and Supplementary Figure 8). Calculated CI means for these combinations suggest more cell death synergy for both VMC40 and P31 cells (Figure 6B), compared to when agents were added to cells at the same time (Figure 5B). In Met-5a cells, adding BGB324 as a pretreatment did not significantly alter the GI_50_ values for cisplatin and pemetrexed. The SRB results for VMC40 and P31 cells were validated using clonogenic assays (Figures 6D,E). The mean CIs from the clonogenic assay were 0.91 for 1.4 μM cisplatin with BGB324 and 0.74 for 0.1 μM pemetrexed with BGB324 in VMC40 cells, suggesting synergistic cytotoxicity. For P31 cells, the mean CIs at concentrations shown were 0.88 for cisplatin with pretreated BGB324 and 0.94 for pemetrexed with pretreated BGB324.

**Figure 7:**
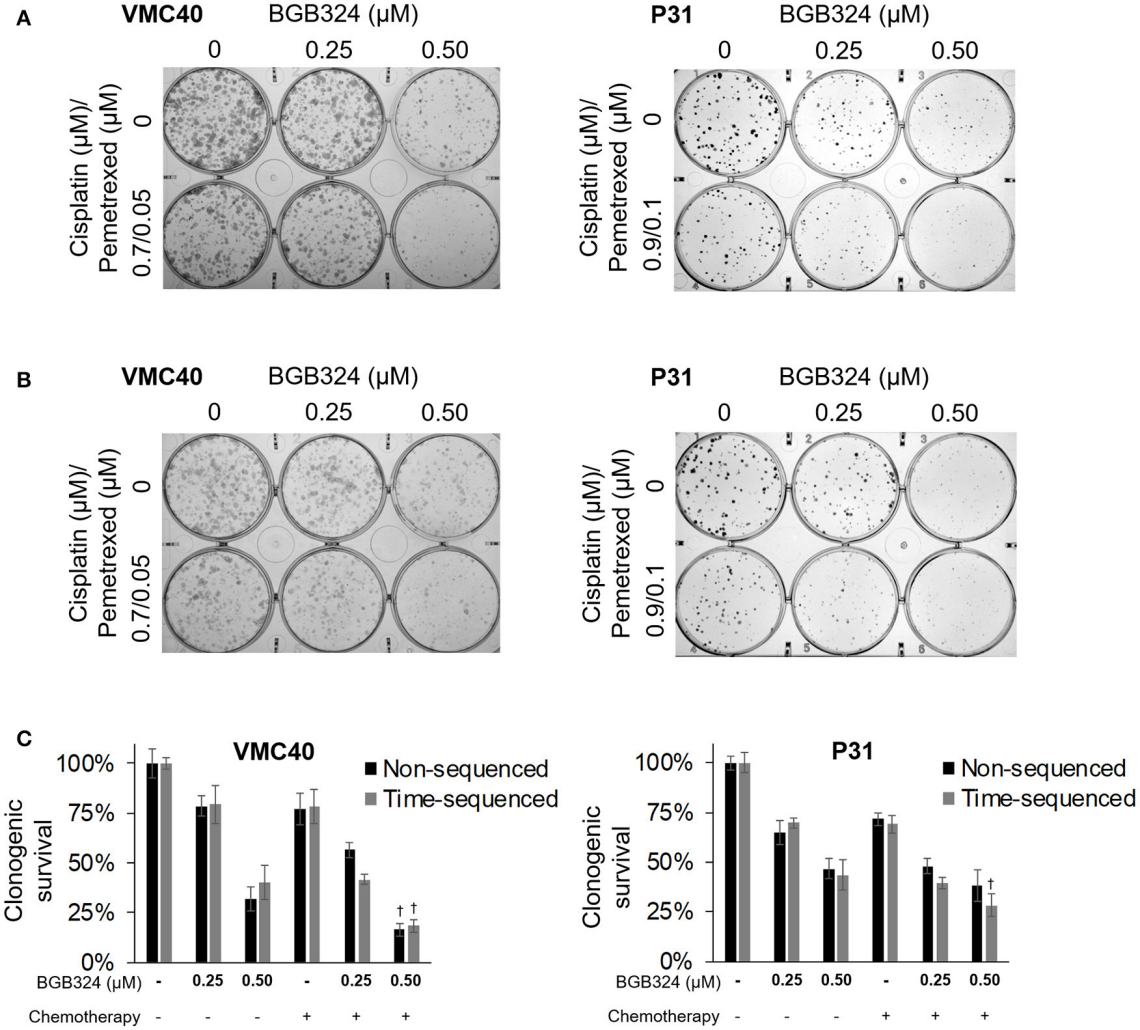
Combination of BGB324 with both cisplatin and pemetrexed. **(A)** Representative images of clonogenic assays after 72 h treatment with BGB324, cisplatin, and pemetrexed. **(B)** Representative images of clonogenic assays after 4-h pretreatment with BGB324 followed 72 h treatment with both cisplatin and pemetrexed. **(C)** Quantification of **(A,B)** by measuring quantity of stain from cells. †Denotes *p* < 0.05 via *t*-test for value compared to values of BGB324 alone. Error bars represent ±SEM of three experiments.

The combination of cisplatin and pemetrexed are the current chemotherapy for MPM (Benedetti et al., 2015; Yap et al., 2017). As a proof-of-concept, we performed clonogenic assays using this combination (at half the concentrations used previously in Figures 5D,E, 6D,E) with BGB324 (Figure 7A). Also, we performed the essentially the same assay with BGB324 being added 4 h prior to chemotherapy agents (Figure 7B). These results were quantified by solubilizing and measuring the stain (Figure 7C). For VMC40 cells, the mean CIs were 0.91 for co-treatment with BGB324 and 0.74 for pretreated with BGB324, indicating synergistic cytotoxic effects. For P31 cells, the mean CIs were 1.04 for co-treatment with BGB324 and 0.92 for pretreatment with BGB324.

## Discussion

Prior evidence of elevated cellular ROS promoting cell survival (Clerkin et al., 2008; Niederst and Engelman, 2013) led to the hypothesis that chemotherapy-induced ROS may also promote these signaling pathways, and inhibiting these pathways may enhance chemotherapy efficacy. In MPM cells, we showed that cisplatin and pemetrexed induce ROS (Figure 2), and phosphorylate AXL and Akt (Figure 3). Moreover, knocking down AXL or blocking AXL activation with BGB324 enhances sensitivity to cisplatin and pemetrexed (Figures 4–7).

**Figure 8:**
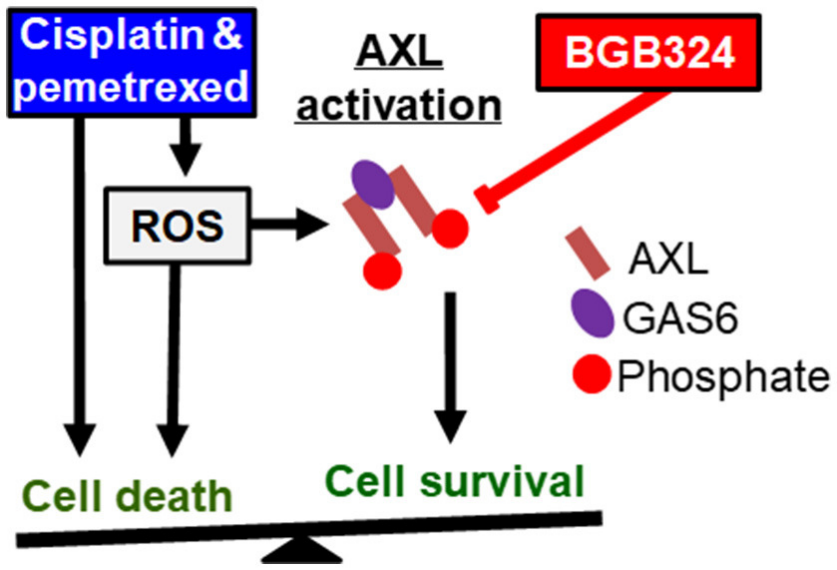
Known pathways and hypothesis for AXL inhibitor (red box) and platinum or antifolate drug (blue box) synergies. Black arrows: Activated AXL promotes cell survival. ROS can increase GAS6 activation and activate AXL independently. Cisplatin and pemetrexed cause cell cycle arrest and increased ROS promoting apoptosis, but ROS-induced AXL activation can inhibit apoptosis. Red line: AXL inhibition can decrease cell survival and promote apoptosis. We have shown adding an AXL inhibitor to current chemotherapy will synergistically promote cell death. This synergy is based on inhibiting an opposing pathway of apoptosis.

Based on these results, we propose a model where the effects of ROS promoting cell survival and apoptosis are in a balance (Figure 8). Cisplatin and pemetrexed both induce ROS generation that contributes to cytotoxicity (Berndtsson et al., 2007; Watson, 2013; Hwang et al., 2015), and ROS levels are elevated in MPM cells after 30 m of treatment with cisplatin or pemetrexed (Figure 2). We showed that these agents induce AXL phosphorylation, which is blocked by the addition of BGB324 inhibitor and N-acetylcysteine antioxidant (Figure 3), suggesting that chemotherapy-induced ROS mediate AXL activation. Cisplatin and pemetrexed also induced phosphorylation of Akt (Figure 3), which may promote cell survival signaling (Clerkin et al., 2008; Hong et al., 2008). We showed that ROS-induced Akt phosphorylation is mediated through AXL because BGB324 attenuates ROS-induced Akt phosphorylation. In addition, we showed that chemotherapy-induced Akt phosphorylation is in part mediated through AXL because BGB324 partially attenuated Akt phosphorylation.

We investigated AXL activation using 150 μM H_2_O_2_, which is a level of ROS consistent with moderate cellular oxidative stress (Ravid et al., 2002; Pal et al., 2007; Oien and Moskovitz, 2008; Oien et al., 2010). For activating AXL using cisplatin and pemetrexed, we used drug concentrations that resulted in similar CM-H2DCFDA fluorescence as 150 μM H_2_O_2_ at 30 m. Using SRB assays, we show pretreatment with 0.5 μM BGB324 can enhance cytotoxicity from 15 μM cisplatin and 8 μM pemetrexed at 72 h (Figures 6A,B, Supplementary Figure 8). Initially, we used the same drug concentrations for 72 h treatment in the clonogenic assay, which resulted in a very minimal quantity of cells producing colonies, even with monotherapy treatment (data not shown). It is interesting that much lower drug concentrations in the clonogenic assay still result in significant cytotoxicity increases when BGB324 is added to cisplatin and pemetrexed (Figures 6D,E). This could indicate cellular ROS is generated by these lower concentrations of cisplatin and pemetrexed over a longer time period, but this still remains to be investigated for both monotherapies and combination therapy.

The Akt pathway can regulate the viability of cells through manipulation of apoptosis-associated molecules or by regulating key apoptosis transcription factors (Clerkin et al., 2008). It is not known if the Akt pathway counteracts ROS-induced apoptosis in our system, but it is likely these opposing pathways are influencing a balance between apoptosis and survival. In support of this concept, we have shown that AXL knockdown enhances sensitivity cisplatin and pemetrexed (Figure 4), suggesting that chemotherapy-induced AXL activation is an adaptive response that may blunt the cytotoxic effect of chemotherapy. Accordingly, AXL inhibitor BGB324 blocks the adaptive response mediated by AXL and enhances sensitivity to cisplatin and pemetrexed (Figure 5). It appears that pretreatment with BGB324 enhances the sensitivity to cisplatin and pemetrexed more than co-treatment with BGB324 (Figure 6), but further investigation is needed to understand the molecular mechanisms contributing to synergism between pre-treatment with BGB324 and chemotherapy. Moreover, it would be important to evaluate the dynamic effects of endogenous antioxidants/antioxidant capacity in this system, especially over longer treatment periods of time and in chemotherapy-resistant cells.

The biochemical mechanism of ROS regulating AXL is unknown. ROS activation of AXL could be direct, through another kinase, or inhibiting an unknown phosphatase (Huang et al., 2013). No direct oxidation of AXL has been reported, however, a similar region in EGFR has been shown to be oxidized with increased phosphorylation (Truong and Carroll, 2012). Furthermore, any direct interaction between AXL and PI3K/Akt has not been reported to our knowledge, and investigation of this pathway may lead to valuable drug target insights.

AXL is activated though autocrine/paracrine signaling by the GAS6 ligand, and this complex is degraded in the lysosome following endocytosis (Wu et al., 2014). The E3 ubiquitin ligase c-Cbl binds AXL, and ubiquitination is required for degradation in the lysosome (Valverde, 2005). AXL is also activated by ROS independent of ligand (Konishi et al., 2004). In the presence of ROS, AXL ubiquitination is reduced, which results in an increased duration of pAXL (Valverde, 2005). Activation of a structurally similar receptor tryrosine kinase, EGFR, in the presence of ROS promotes accumulation on the cell membrane, but this effect has not been studied for AXL (Ravid et al., 2002). EGFR is aberrantly phosphorylated and degradation is reduced in the presence of hydrogen peroxide (Filosto et al., 2011). Evidence also supports that cysteine oxidation in the ATP-binding region promotes phosphorylation of EGFR (Truong and Carroll, 2012). Moreover, IGF1R is rapidly expressed and activated in the presence of hydrogen peroxide, and inhibition of this receptor has been shown to promote apoptosis (Azar et al., 2007; Baregamian et al., 2012). Several reports of AXL phosphorylation have demonstrated this to be fast and quickly degraded (Valverde, 2005; Bae et al., 2015). Therefore, changes in redox biology induced by chemotherapy likely contribute to AXL activation and adaptive response mediated by AXL.

Adaptive cellular response to chemotherapy plays an important role in providing the cells with transient resistance to chemotherapy and may ultimately contribute to the acquired chemotherapy resistance (Goldman et al., 2015). Therefore, our discovery of AXL-mediated adaptive response and our finding that AXL inhibition enhances sensitivity to chemotherapy is significant. In summary, we show here that cisplatin and pemetrexed can activate AXL, which can be blocked by the BGB324 AXL inhibitor. Adding BGB324, especially as a pretreatment, can enhance the cell death efficacy of cisplatin and pemetrexed in MPM cells. These studies may support future *in vivo* investigations on the ability of BGB324 to enhance cisplatin and pemetrexed tumor reduction.

## Author contributions

All authors were involved in the initial development of this project and editing this manuscript. TG planned and oversaw the RPPA analysis. SE ran pilot studies and assay optimization for the use of BGB324. DO and JC were responsible for the hypothesis and experimental completion of the project. DO designed and executed all the other experiments, and wrote this manuscript.

### Conflict of interest statement

The authors declare that the research was conducted in the absence of any commercial or financial relationships that could be construed as a potential conflict of interest.
