# Supplementary material for: Cisplatin and Pemetrexed Activate AXL and AXL Inhibitor BGB324 Enhances Mesothelioma Cell Death from Chemotherapy
Source: Front Pharmacol. 2018 Jan 11;8:970. doi: 10.3389/fphar.2017.00970 (PMC5768913; doi:10.3389/fphar.2017.00970)
Supplement: Supplementary file 1 [file DataSheet1.DOCX]

Supplementary Material

Cisplatin and pemetrexed activate AXL and AXL inhibitor BGB324 enhances mesothelioma cell death from chemotherapy

Derek B. Oien^1^, Tamas Garay^2^, Sarah Eckstein^1^, Jeremy Chien^1*^

*** Correspondence:** Jeremy Chien, jchien@salud.unm.edu

## Supplementary Figures


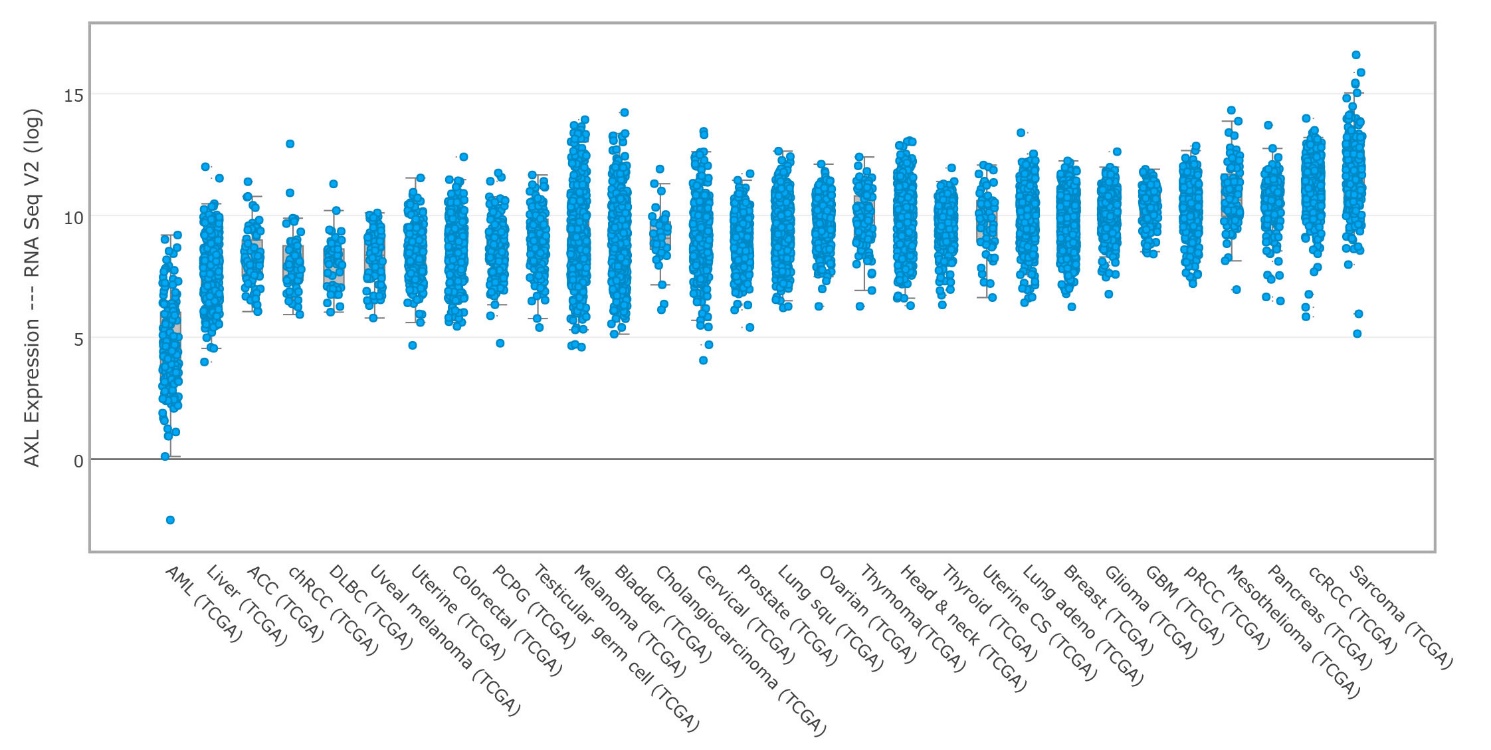


**Supplementary Figure 1.** AXL expression in clinical samples. In a total of 30 TCGA cancer datasets, MPM (87 samples, blue dots) ranked fourth highest for AXL expression. Data are RNA Seq V2 values from TCGA Research Network, represented by graphs generated at cBioPortal ([1](#_ENREF_1), [2](#_ENREF_2)).

**
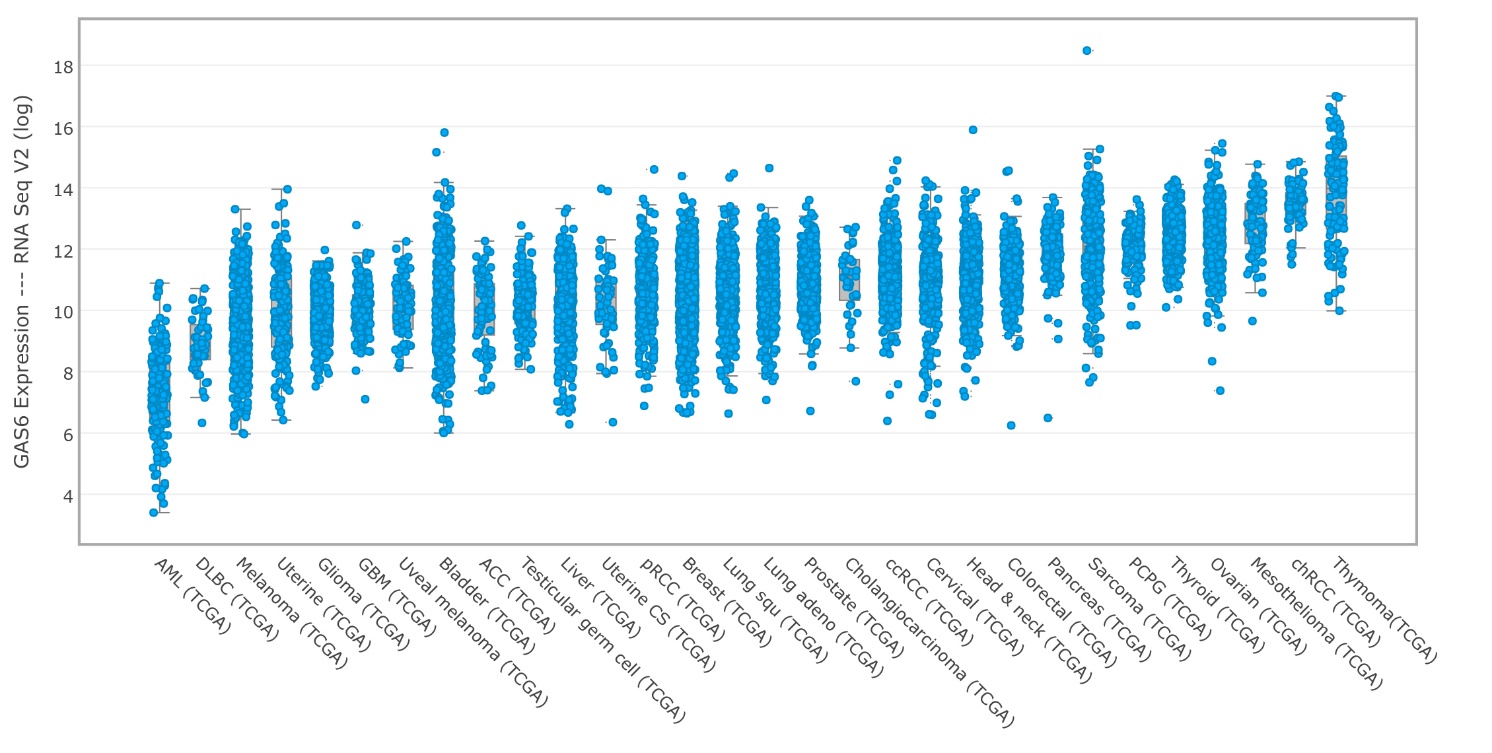
**

**Supplementary Figure 2.** GAS6 expression in clinical samples. In a total of 30 TCGA cancer datasets, MPM (87 samples, blue dots) ranked third highest for GAS6 expression. Data are RNA Seq V2 values from TCGA Research Network, represented by graphs generated at cBioPortal ([1](#_ENREF_1), [2](#_ENREF_2)).

**Supplementary Figure 3.** Reverse phase protein heat map for MPM cell lines. Cell extracts applied to coated slides and probed with 301 antibodies, normalized to loading. Color brightness indicates protein detection relative to median, *red* for increased and *green* for decreased detection levels. For example, normalized log_2_ median-centered value of AXL was 1.35 for VMC40. Antibody status indicated V for validated antibody, C for validation in progress, Q for possible nonspecific signal, E for under evaluation, M for mouse, G for goat, R for rabbit, and T for Rat.

**Supplementary Figure 4.** A) Quantification of pAXL and pAkt from immunoblots in Figure 3. *Error* *bars* represent ±SEM of three experiments. * and † denote *p* < 0.05 via *t*-test for value compared to untreated control and to addition of BGB324, respectively. B) Effect of BGB324 in the absence of exogenous ROS and chemotherapy agent. Cells were treated with 2 µM BGB324 at time indicated and compared to no treatment. Positive controls were 150 µM hydrogen peroxide.

**Supplementary Figure 5.** Comparison at specified drug concentrations for SRB cell death values in Figure 4C. *Error* *bars* represent ±SEM of three experiments. † denotes *p* < 0.05 via *t*-test for value compared to NTC at specified drug concentration.

**Supplementary Figure 6.** Representative clonogenic assay images of AXL shRNA-transfected cell lines with cisplatin and pemetrexed using incrementing concentrations as indicated. Cells were stained with SRB for visualization.

**Supplementary Figure 7.** A-B) Short-term cell survival values for mesothelioma cells exposed to combinations of (A) cisplatin or (B) pemetrexed agents and BGB324 at concentrations indicated. Value represents percentage cell survival. Single agent treatment in *blue boxes*. Each value represents mean of three experiments. Cisplatin at 30 µM shown for reference, not a clinically relevant concentration. C) Graphical representation of cell survival values for BGB324 concentrations in (A-B) and with 4 µM BGB324 (omitted from CI analysis based on cell death < 20% for VMC40). D) Comparison at specified drug concentrations for SRB cell death values in Figure 5A. BGB324 concentration was 0.5 µM. *Error* *bars* represent ±SEM of three experiments. † denotes *p* < 0.05 via *t*-test for value compared to absence of BGB324 at specified drug concentration.

**Supplementary Figure 8.** A-B) Short-term cell survival values for mesothelioma cells exposed to combinations of (A) cisplatin or (B) pemetrexed agents and 4-h pretreatment with BGB324 at concentrations indicated. Value represents percentage cell survival. Single agent treatment in *blue boxes*. Each value represents mean of three experiments. Cisplatin at 30 µM shown for reference, not a clinically relevant concentration. C) Comparison at specified drug concentrations for SRB cell death values in Figure 6A. BGB324 was added as 4-h pretreatment and concentration was 0.5 µM. *Error* *bars* represent ±SEM of three experiments. † denotes *p* < 0.05 via *t*-test for value compared to absence of BGB324 at specified drug concentration.

**Supplementary References**

1. Cerami E, Gao J, Dogrusoz U, Gross BE, Sumer SO, Aksoy BA, et al. The cBio cancer genomics portal: an open platform for exploring multidimensional cancer genomics data. *Cancer discovery* (2012) **2**(5):401-4. Epub 2012/05/17. doi: 10.1158/2159-8290.cd-12-0095. PubMed PMID: 22588877; PubMed Central PMCID: PMCPmc3956037.

2. Gao J, Aksoy BA, Dogrusoz U, Dresdner G, Gross B, Sumer SO, et al. Integrative analysis of complex cancer genomics and clinical profiles using the cBioPortal. *Science signaling* (2013) **6**(269):pl1. Epub 2013/04/04. doi: 10.1126/scisignal.2004088. PubMed PMID: 23550210; PubMed Central PMCID: PMCPmc4160307.
